# Development and validation of nomogram prognostic model for predicting OS in patients with diffuse large B-cell lymphoma: a cohort study in China

**DOI:** 10.1007/s00277-023-05418-9

**Published:** 2023-08-24

**Authors:** Xiaosheng Li, Qianjie Xu, Cuie Gao, Zailin Yang, Jieping Li, Anlong Sun, Ying Wang, Haike Lei

**Affiliations:** 1 Chongqing Cancer Multi-omics Big Data Application Engineering Research Center, Chongqing University Cancer Hospital, Chongqing, 400030 China; 2 Department of Health Statistics, School of Public Health, Chongqing Medical University, Chongqing, 400016 China

**Keywords:** Diffuse large b-cell lymphoma (DLBCL), Non-Hodgkin’s lymphoma, Nomogram model, Prognosis, Overall survival

## Abstract

This study comprehensively incorporates pathological parameters and novel clinical prognostic factors from the international prognostic index (IPI) to develop a nomogram prognostic model for overall survival in patients with diffuse large B-cell lymphoma (DLBCL). The aim is to facilitate personalized treatment and management strategies. This study enrolled a total of 783 cases for analysis. LASSO regression and stepwise multivariate COX regression were employed to identify significant variables and build a nomogram model. The calibration curve, receiver operating characteristic (ROC) curve, and decision curve analysis (DCA) curve were utilized to assess the model’s performance and effectiveness. Additionally, the time-dependent concordance index (C-index) and time-dependent area under the ROC curve (AUC) were computed to validate the model’s stability across different time points. The study utilized 8 selected clinical features as predictors to develop a nomogram model for predicting the overall survival of DLBCL patients. The model exhibited robust generalization ability with an AUC exceeding 0.7 at 1, 3, and 5 years. The calibration curve displayed evenly distributed points on both sides of the diagonal, and the slopes of the three calibration curves were close to 1 and statistically significant, indicating high prediction accuracy of the model. Furthermore, the model demonstrated valuable clinical significance and holds the potential for widespread adoption in clinical practice. The novel prognostic model developed for DLBCL patients incorporates readily accessible clinical parameters, resulting in significantly enhanced prediction accuracy and performance. Moreover, the study’s use of a continuous general cohort, as opposed to clinical trials, makes it more representative of the broader lymphoma patient population, thus increasing its applicability in routine clinical care.

## Introduction

Diffuse large B-cell lymphoma (DLBCL) is a highly heterogeneous malignant tumor arising from B lymphocytes, characterized by diffuse growth. It represents the most prevalent subtype of non-Hodgkin’s lymphoma, with a global prevalence of approximately 40%, increasing with age. The clinical and prognostic diversity in DLBCL is remarkably high [1, 2]. While pre-chemotherapy immunotherapy achieves cure in most patients, approximately 50% will experience relapse, and one-third will succumb to the disease [3]. Therefore, it is crucial to develop risk prediction models for early risk stratification and optimal treatment decisions.

Historically, the International Prognostic Index (IPI) has been commonly employed to provide straightforward and reliable prognostic information to newly diagnosed DLBCL patients. It utilizes clinicopathological parameters such as age, lactate dehydrogenase (LDH) levels, number/location of involvement, disease stage, and patient performance status (PS) to predict treatment response and prognosis [4]. While IPI became the gold standard for non-Hodgkin lymphoma risk classification, its ability to fully explain the disease’s heterogeneity remains limited [5]. The addition of rituximab to the traditional regimen in the late 1990s significantly improved survival rates across all risk groups, reducing IPI’s effectiveness in risk stratification, especially for high-risk patients [6]. Consequently, the revised IPI (R-IPI) and National Comprehensive Cancer Network IPI (NCCN-IPI) were introduced as optimized versions for adequate risk stratification in DLBCL [7]. Nevertheless, these models still have shortcomings. R-IPI is based on a cohort of only a few patients over 60 years old, potentially limiting its value for elderly patients [8]. While NCCN-IPI is considered useful, the extranodal organs used in this indicator may not consistently indicate poor prognosis. Additionally, NCCN-IPI’s repeatability is questioned in cases involving positron emission tomography (PET)-computer tomography (CT) staging [9]. Moreover, studies have shown that R-IPI and NCCN-IPI have lower predictive power for low-risk groups [10]. Advancements in medical technology and an increasing array of DLBCL treatments have led to the identification of new clinical variables as potential prognostic markers. However, IPI, R-IPI, and NCCN-IPI have not incorporated these novel clinical prognostic factors.

The nomogram is a powerful graphical tool or algorithm that integrates both biological and clinical variables. It plays a crucial role in clinical decision-making and risk stratification within the field of oncology [11]. In current clinical research, nomograms are extensively used for predicting individual prognosis [12, 13].

The objective of this study is to construct a nomogram prognostic model for the overall survival of DLBCL patients. The model considers a wide range of clinical characteristics, incorporating pathological parameters and new clinical prognostic factors from the IPI. The ultimate aim is to provide guidance for personalized treatment and management strategies.

## Materials and methods

### Patient characteristics and study design

This study is a prospective cohort study that collected data from all newly diagnosed DLBCL patients between January 1, 2013, and December 31, 2018, at Chongqing University Cancer Hospital, resulting in a total of 894 cases. The following information was recorded: patient-related factors, including gender, age, ethnicity, marital status, medical payment method, discharge method, and B symptoms; cancer-related factors, such as Ann Arbor Stage; treatment-related factors, encompassing chemotherapy, radiotherapy, targeted therapy, immunotherapy, and surgery; biomarkers, including lactate dehydrogenase (LDH), β2-microglobulin (β2-M), CD4/CD8 ratio, albumin/globulin ratio (A/G), platelet count (PLT), lymphocyte count (LYM), and C-reactive protein (CRP). It is important to note that due to various reasons, a significant number of individuals in China may opt for Chinese medicine treatment or conservative treatment, making it challenging to collect and include treatment information for these patients in the hospital database. Consequently, this study did not take into account traditional Chinese medicine treatment or conservative treatment for patients. All laboratory tests were conducted in the laboratory of Chongqing University Cancer Hospital.

### Inclusion and exclusion criteria

**Fig. 1:**
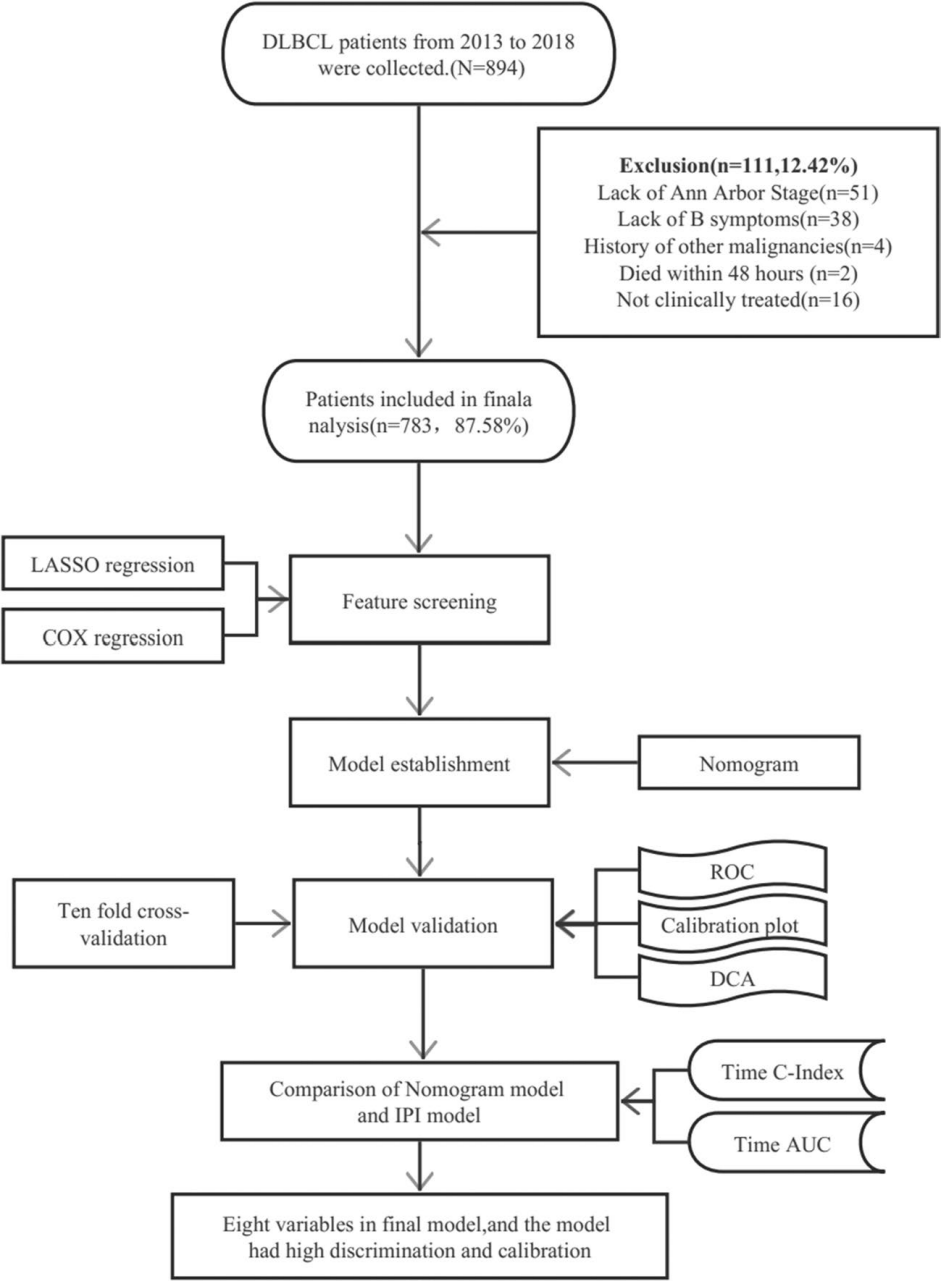
Flow diagram of study design

The study’s inclusion criteria were as follows: [1] age ≥ 18 years; [2] documentation of at least one hospitalization; [3] newly diagnosed DLBCL confirmed by pathological examination; [4] no previous history of other malignant tumors; [5] completion of major clinical treatment within the hospital. Exclusion criteria included: [1] death within 48 h after admission; [2] significant missing and incomplete clinical data (e.g., major treatment and examination information data). The study’s flowchart is illustrated in Fig. 1. This research was conducted in accordance with the guidelines outlined in the Declaration of Helsinki and received approval from the Ethics Committee of Chongqing University Cancer Hospital. Written informed consent was obtained from all participants.

### Construction and validation of the nomogram

We developed and validated a nomogram model to assess the prognosis of DLBCL patients using a dataset of 783 patients who met the inclusion and exclusion criteria. To create an efficient clinical prognostic model, we optimized the collected data and carefully selected the most relevant features as predictors to strike the best balance between model performance and clinical applicability. In the process of feature selection, we utilized LASSO (least absolute shrinkage and selection operator) regression to screen the clinical features in the training cohort. The optimal parameter (*λ*) for LASSO regression was determined through cross-validation, and the essential variables were selected based on the principle of minimum *λ*. Subsequently, we employed stepwise multivariate COX regression on these variables, further refining the selection based on the principle of minimum AIC (Akaike information criterion). This two-step variable screening method enabled us to gain deeper insights into the data and reduce model complexity. Moreover, we considered the clinical significance of these variables during the screening process, not solely relying on statistical significance. Ultimately, we identified 8 variables as predictors: age, Ann Arbor stage, LDH, β2-microglobulin, CD4/CD8 ratio, LYM, CRP, and B symptoms. Based on the results of the Cox regression model, we constructed a nomogram to predict the probabilities of 1-, 3-, and 5-year overall survival (OS).

To evaluate the model’s performance, we employed a series of graphical tools. The calibration curve assessed the prediction accuracy and discrimination ability of the model [14]. The receiver operating characteristic (ROC) curve confirmed the model’s generalization ability [14]. The decision curve analysis (DCA) was used to determine the clinical significance of the model [15].The concordance index (C-index) served as a commonly used measure of model performance [16]. First proposed by Professor Frank E Harrell Jr., a biostatistician at Vanderbilt University in 1996 [17], the C-index gauges the concordance between the COX model’s predicted values and the actual outcomes in survival analysis. It calculates the proportion of patient pairs in which the predicted and observed outcomes agree. Additionally, we conducted 100 10-fold cross-validations and computed the average C-index and average AUC (area under the curve) to evaluate the nomogram model. The C-index and AUC evaluated at different time points are referred to as time-dependent C-index [18] and time-dependent AUC [19]. Time-dependent C-index assesses the consistency between the order of death and the specified risk level in the queue. Time-dependent AUC measures the accuracy of determining a patient’s status at time *t*, indicating whether patients who survive at time *t* have a higher predicted survival probability compared to those who die [20]. These measures were employed to verify the accuracy and robustness of the model at various time intervals.

In conclusion, this study utilized a rigorous methodology to develop and validate a nomogram model for DLBCL patients, ensuring its clinical relevance and predictive power.

### Model comparison

As a well-established prognostic tool for DLBCL patients widely used in clinical practice, the International Prognostic Index (IPI) staging has consistently demonstrated excellent predictive accuracy and robustness over the years [21]. To further evaluate the performance of the nomogram model developed in this study, we employed two additional indices, namely, the net reclassification improvement (NRI) and the integrated discrimination improvement (IDI). The NRI is a measure used to compare the prediction ability of two models. It assesses the improvement in prediction accuracy of the new model compared to the reference model [22]. NRI pays particular attention to the relative advantages and disadvantages of the two models at a specific cutoff value, providing valuable insights into their respective strengths. On the other hand, IDI is commonly used to reflect the overall improvement of the model. It measures the enhancement of risk prediction ability in the new model relative to the reference model [23]. By considering the overall improvement, IDI provides a more comprehensive evaluation of the model’s performance.

In this study, we constructed an IPI model using the IPI of tumors as a reference and subsequently compared the advantages and disadvantages of the Nomogram model and the classical IPI model from four perspectives: time-dependent C-index, time-dependent AUC, NRI, and IDI. By incorporating NRI and IDI into the evaluation, we aim to provide a more comprehensive assessment of the nomogram model’s predictive capabilities, offering valuable clinical interpretation and insights for decision-making in DLBCL patient management.

### Statistical analysis

The primary outcome of this study was to determine the probability of 1-, 3-, and 5-year overall survival (OS) for DLBCL patients. OS was defined as the time from the initial diagnosis to death, loss to follow-up, or the last follow-up. Follow-up assessments were conducted every 3–6 months during the first 2 years after diagnosis, followed by annual follow-ups until death. The study was censored in April 2022, and the follow-up period was measured in months.

To handle missing data, the mice 3.0.0 package (Van Buuren and Groothuis-Oudshoorn, 2011) was employed. This package employs a method to impute missing data, enhancing accuracy and statistical power compared to other missing data techniques. Multiple imputations (replacement values) for multivariate missing data are created based on the fully conditional specification method, where each incomplete variable is imputed using a separate model. The multivariate imputation via chained equations (MICE) algorithm can impute a mix of continuous, binary, unordered categorical, and ordered categorical data [24]. Furthermore, MICE can handle imputations for continuous two-level data while maintaining consistency between imputations through passive imputation [25].

Enumeration data were described using frequency and percentage and compared using the chi-square test. Continuous data that did not follow a normal distribution were described using the median and interquartile range (IQR) and compared using nonparametric tests. The nomogram model was constructed, and the C-index was calculated using the survival package (version 3.3-1). Various graphs in the study were generated using packages such as pROC (version 1.8.0), ggDCA (version 1.2), timeROC (version 0.4), and pec (version 2022.5.04.04). The tenfold cross-validation was performed using the caret package (version 6.0-92). An online calculator based on the nomogram was developed using the shiny (version 1.7.1) and DynNom packages (version 5.0.1) for individual and dynamic predictions of patient survival rates. The statistical analyses were conducted using R (version 4.2.1; https://www.r-project.org/) and RStudio (version 2022.07.1-554; https://rstudio.com/products/rstudio/). Statistical significance was considered when *P* < 0.05.

## Results

### Characteristics of subjects

Table 1 presents the clinical features of 783 lymphoma patients included in this study. The median age of the patients was 59, with significantly older age observed in patients who succumbed to the disease compared to those who survived (62 vs. 56, *P* < 0.05). There was no significant difference in mortality observed at different levels of platelet count (PLT) and B symptoms. Notably, patients who received chemotherapy and targeted therapy exhibited significantly lower mortality rates (*P* < 0.05). However, it is worth mentioning that the number of individuals receiving treatment is smaller than those not receiving treatment. This discrepancy is attributed to the exclusion of traditional Chinese medicine treatment or conservative treatment received by patients outside the hospital. In reality, nearly every patient received varying degrees of treatment. Among the laboratory parameters, deceased patients displayed higher levels of LDH and β2-M and lower levels of CD4/CD8, A/G, and lymphocytes (LYM) (*P* < 0.05). Furthermore, 97.19% of patients did not exhibit B symptoms during the course of the study.

**Table 1:** Clinical characteristics of the patients

Item	Overall	Survival (*n* = 371)	Death (*n* = 412)	*P*-value
Medical insurance (%)				
Residents	432	193 (44.68)	239 (55.32)	0.0049^a^
Employees	234	131 (55.98)	103 (44.02)	
Others	117	47 (40.17)	70 (59.83)	
Ann Arbor Stage (%)				
I-II	282	173 (61.35)	109 (38.65)	< 0.0001^a^
III	212	99 (46.7)	113 (53.3)	
IV	289	99 (34.26)	190 (65.74)	
Chemotherapy (%)				
No	545	222 (40.73)	323 (59.27)	< 0.0001^a^
Yes	238	149 (62.61)	89 (37.39)	
Targeted therapy (%)				
No	444	171 (38.51)	273 (61.49)	< 0.0001^a^
Yes	339	200 (59)	139 (41)	
B symptoms (%)				
No	761	365 (47.96)	396 (52.04)	0.0892^a^
Yes	22	6 (27.27)	16 (72.73)	
Age (median [IQR])	59.00 [48.00, 68.00]	56.00 [46.00, 65.00]	62.00 [51.00, 70.00]	< 0.0001^b^
LDH (median [IQR])	228.20 [179.05, 342.50]	204.10 [166.95, 261.70]	270.95 [195.90, 432.85]	< 0.0001^b^
β2-M (median [IQR])	2.50 [1.80, 3.50]	2.15 [1.70, 2.90]	2.99[2.10, 4.33]	< 0.0001^b^
CD4/CD8 (median [IQR])	1.31 [0.90, 1.81]	1.39 [0.97, 1.91]	1.26 [0.80, 1.71]	0.0041^b^
A/G (median [IQR])	1.43 [1.18, 1.66]	1.47 [1.23, 1.70]	1.38 [1.12, 1.61]	0.0001^b^
PLT (median [IQR])	197.00 [147.00, 256.00]	200.00 [150.00, 255.00]	195.00 [145.00, 259.25]	0.6278^b^
LYM (median [IQR])	1.28 [0.93, 1.68]	1.35 [1.00, 1.73]	1.20 [0.85, 1.63]	0.0104^b^
CRP (median [IQR])	5.00 [5.00, 9.22]	5.00 [5.00, 5.00]	5.00 [5.00, 50.57]	< 0.0001^b^

^a^*P* value is obtained by chi-square test

^b^*P* value is obtained by nonparametric test

### Construction of the prognostic nomogram for OS

**Fig. 2:**
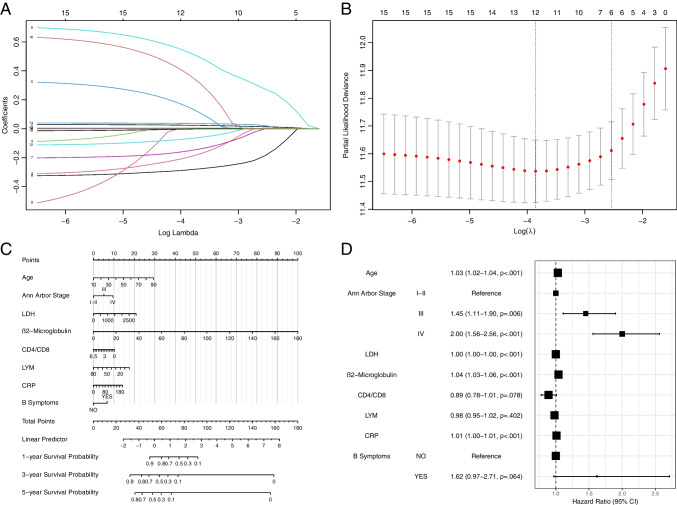
Results of the prognostic nomogram for OS: **A** Construction of a predictive model of lymphoma in the dataset by LASSO regression. **B** Each curve in the figure represents the changed trajectory of each independent variable coefficient. **C** Nomogram for predicting the 1-, 3-, and 5-year OS of lymphoma patients. **D** The hazard ratio plot of stepwise multivariate COX regression

After conducting a trend test for Schoenfeld residuals, it was determined that the data in this study met the proportional risk hypothesis of COX regression. Following two steps of variable screening, the variables age, Ann Arbor Stage, LDH, β2-microglobulin, CD4/CD8, LYM, CRP, and B symptoms were retained based on their multivariate COX regression results (Fig. 2A, B). Using this information, a nomogram model was established for predicting the 1-, 3-, and 5-year overall survival (OS) prognosis of DLBCL patients (Fig. 2C).

The nomogram allows for calculating the total number of points per patient based on the assigned points for each risk variable. A higher score on the nomogram indicates a worse prognosis. For instance, if a patient is 74 years old, with Ann Arbor Stage IV, LDH level of 322.0, β2-microglobulin level of 2.30, CD4/CD8 ratio of 1.53, LYM count of 1.34, CRP level of 5.00, and without B symptoms, the total score on the model prediction would be 74.73. The 1-year survival probability for this patient would be 0.7699, the 3-year survival probability would be 0.3966, and the 5-year survival probability would be 0.2389. This indicates that the patient may have a good quality of life in the short term, but the long-term survival probability is concerning, and additional treatment considerations may be required during clinical management. Furthermore, an online calculator based on the nomogram (https://dlbcl.shinyapps.io/DynNomapp/) was developed to predict long-term OS in DLBCL patients.

In the nomogram model, age was identified as a risk factor for OS, with a hazard ratio (HR) of 1.03 (*P* < 0.001). This means that for every year of age, the risk of death increases by 3%. Similarly, Ann Arbor Stage IV, β2-microglobulin, and CRP were also identified as risk factors for OS. Compared to patients with stage I–II, the risk of death for patients with Ann Arbor Stage IV increased by 100% (*P* < 0.001). For every unit increase in β2-microglobulin and CRP, the risk of death in DLBCL patients increased by 4% and 1%, respectively (Fig. 2D).

### Performance of the nomogram model

**Fig. 3:**
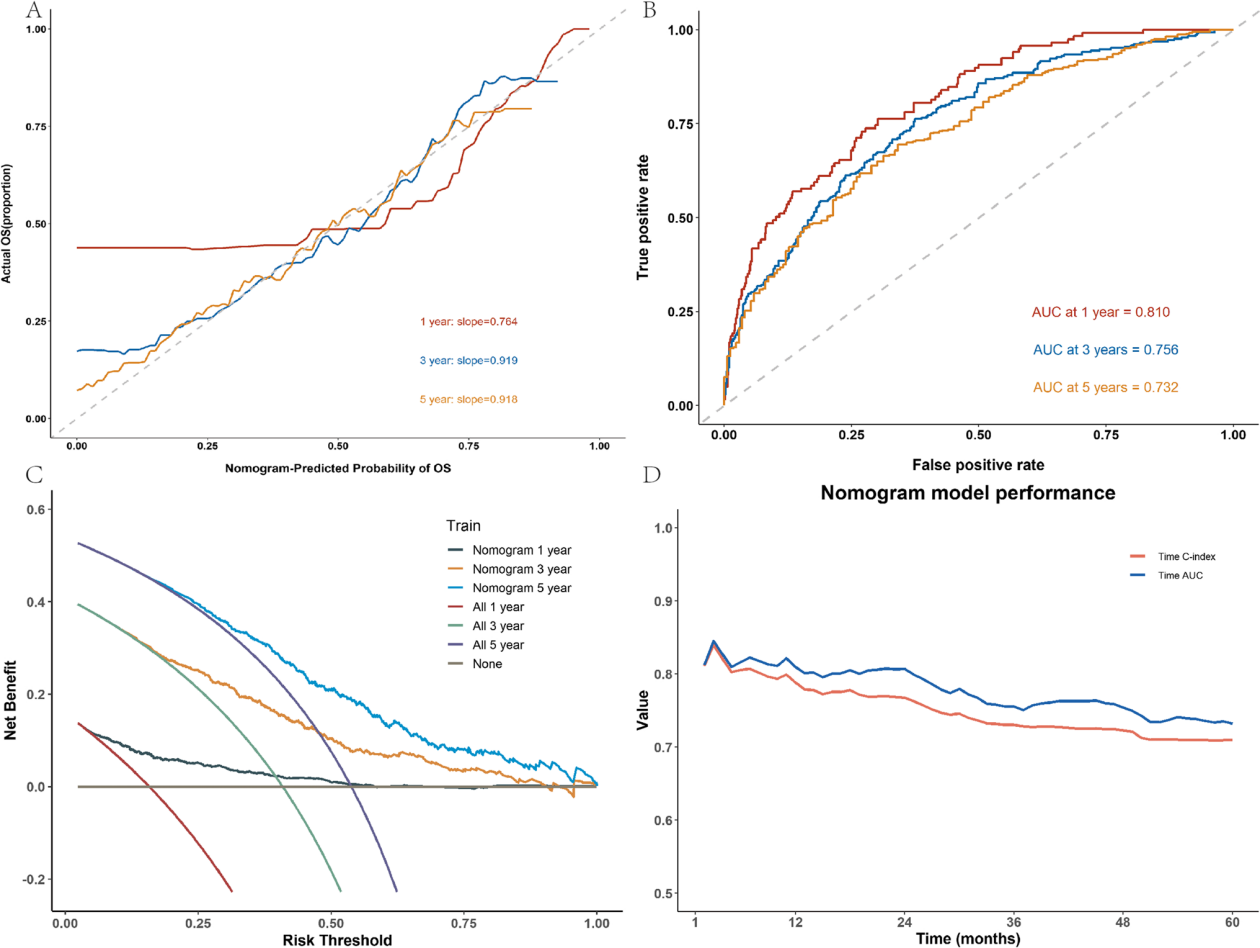
Nomogram model effect. **A** 1-, 3-, and 5-year calibration curves of nomogram model; **B** 1-, 3-, and 5-year ROC curves for nomogram models; **C** 1-, 3-, and 5-year DCA curves for nomogram models; **D** time-dependent C-index and time-dependent AUC of nomogram model

Figure 3A displays the calibration curve for all the data. The slopes of the calibration curves for 1-, 3-, and 5-years were 0.764, 0.919, and 0.918, respectively, and all were statistically significant (*P* < 0.05). The distribution of points on both sides of the diagonal line is even, indicating that the nomogram model exhibits excellent prediction accuracy. Additionally, the C-index(se) for the data was calculated as 0.73 (0.0123), further confirming the high accuracy of the model. Figure 3B illustrates the ROC curves of the nomogram model for 1, 3, and 5 years. The AUC values for all the curves in the graph exceeded 0.7, indicating the outstanding generalization ability of the nomogram model. Figure 3C emphasizes the high clinical significance of the nomogram model in practical applications. Furthermore, we conducted 100 iterations of tenfold cross-validation to calculate the average C-index and AUC of the model, ensuring its stability. The average C-index(se) for the model was 0.72 (0.0040), and the average AUC (se) was 0.81 (0.0067). These results validate the robustness of the model.

These findings collectively demonstrate that the nomogram model exhibits excellent prediction accuracy, generalization ability, and stability, making it a valuable tool in clinical practice. To investigate the potential impact of time on the model’s performance, this study calculated the time-dependent C-index and time-dependent AUC, as depicted in Fig. 3D. The curve in Fig. 3D demonstrates no significant fluctuations or discernible linear trends, suggesting that the C-index and AUC values of the nomogram model remain relatively stable across the study’s time range. This indicates that the model exhibits good robustness and is capable of accurately predicting the overall survival (OS) of DLBCL patients.

### Comparison with IPI model

**Fig. 4:**
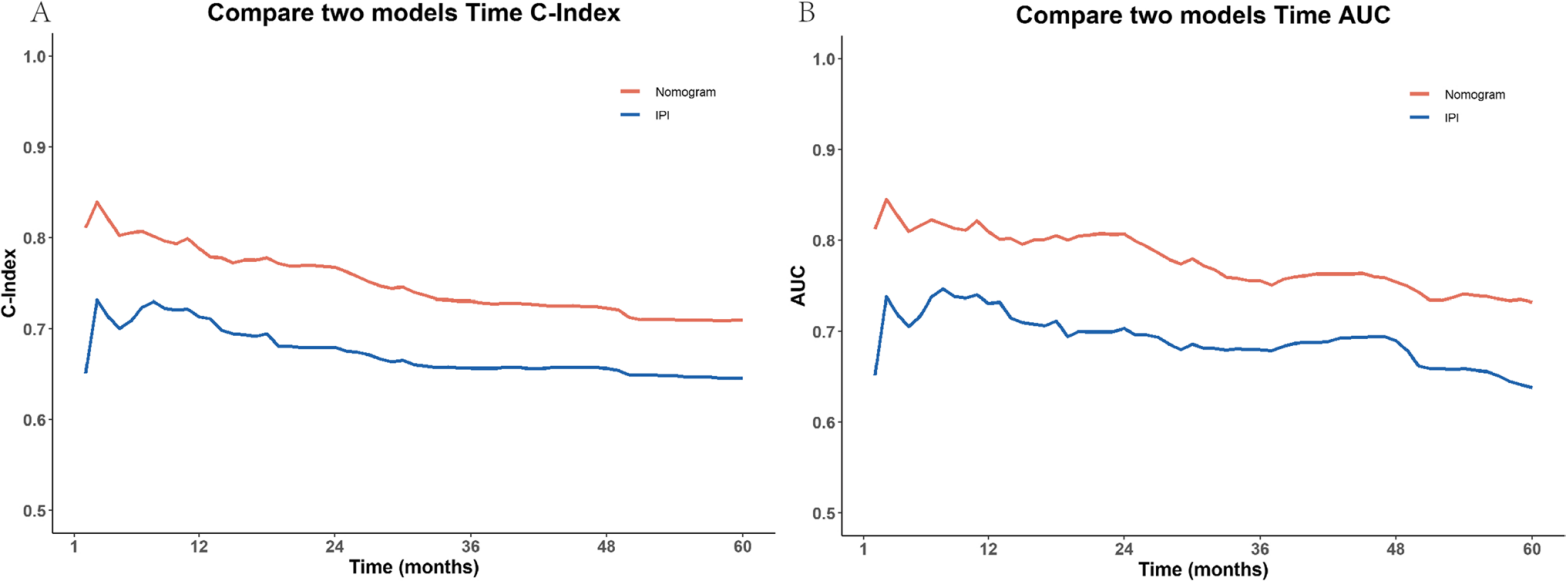
Comparison of nomogram model and IPI model. **A** Time-dependent C-index; **B** time-dependent AUC

To assess the superiority of the nomogram model developed in this study over widely used prognostic models for DLBCL in clinical practice, we compared it with the continuous form of the International Prognostic Index (IPI) model. Table 2 presents a comparison of the nomogram model and the IPI model in terms of C-index and 1-, 3-, and 5-year AUC. Figure 4 illustrates the time-dependent C-index and time-dependent AUC of the Nomogram model and the IPI model, allowing for an evaluation of their respective performance.

**Table 2:** Performance measures for nomogram model and IPI model

Model	C-index (se)	1 year AUC (95%CI)	3 year AUC (95%CI)	5 year AUC (95%CI)
Nomogram	0.73 (0.0123)	0.810 (0.769, 0.850)	0.756 (0.720, 0.792)	0.732 (0.688, 0.777)
IPI model	0.65 (0.0135)	0.73 (0.686, 0.776)	0.68 (0.642, 0.718)	0.64 (0.590, 0.686)

The results clearly indicate that the nomogram model constructed in this study outperforms the IPI model in terms of predictive ability. It exhibits superior performance and significantly better predictive accuracy compared to the IPI model. Furthermore, the 1-, 3-, and 5-year net reclassification improvement (NRI) values for the two models were 0.3389, 0.2473, and 0.0731, respectively. This means that the Nomogram model improved the predictive ability of the TNM staging model by 33.89%, 24.73%, and 7.31% for 1-, 3-, and 5-year predictions, respectively. Additionally, the 1-, 3-, and 5-year integrated discrimination improvement (IDI) values for the two models were 0.092, 0.090, and 0.096, respectively. These findings indicate that the nomogram model outperformed the IPI staging model by 9.2%, 9.0%, and 9.6% in terms of performance for 1-, 3-, and 5-year predictions, respectively.

Consequently, the nomogram model developed in this study exhibits promising potential for clinical application and holds great value in improving prognostic predictions for DLBCL patients.

## Discussion

In this study, we have successfully developed a prognostic prediction model for overall survival (OS) in DLBCL patients using a combination of multiple clinical parameters. The International Prognostic Index (IPI) has been widely utilized as a scoring system for evaluating the prognosis of DLBCL over the past 25 years. However, with advancements in medical technology, it has become apparent that the simple clinical parameters used in IPI may not fully capture the diverse characteristics of patients [26]. To address the limitations of IPI, alternative models such as R-IPI and NCCN-IPI have been proposed. Biccler et al. reported that although the R-IPI model is simpler, it exhibits lower prediction accuracy compared to the IPI and NCCN-IPI models, which demonstrate higher predictive capabilities [27]. It is well-established that Ann Arbor Stage is a critical prognostic factor for DLBCL [28]. However, relying solely on Ann Arbor Stage to predict patient prognosis may not be optimal. Instead, incorporating Ann Arbor Stage into a comprehensive model can significantly enhance the model’s performance [29].

In light of these observations, our newly developed nomogram model takes into account a variety of clinical parameters, including age, LDH, β2-microglobulin, CD4/CD8 ratio, LYM count, CRP, and the presence of B symptoms. By integrating these factors, our model provides a more accurate and robust prediction of the OS of DLBCL patients, offering improved clinical utility and personalized treatment guidance. Indeed, the development of a new prognostic nomogram model for DLBCL, which utilizes more common and representative clinical variables along with better available clinical data in modern medicine, holds great promise for widespread future use. This study not only incorporates traditional clinical features from the IPI, such as age, LDH, and Ann Arbor Stage but also includes additional essential clinical variables, such as CD4/CD8, β2-microglobulin, LYM, CRP, and B symptoms, to construct a comprehensive and advanced prognostic nomogram model for DLBCL. The high C-index of 0.73 for the model demonstrates its strong predictive ability. Furthermore, the development of an online calculator based on the nomogram enhances its convenience for clinicians to dynamically predict the OS rate of DLBCL patients at various time points. This online tool will facilitate more precise risk stratification and personalized treatment decisions, leading to improved patient outcomes and better management of DLBCL. Overall, the new prognostic nomogram model presented in this study represents a significant advancement in DLBCL prognostication, combining the power of modern medical data and technology to enhance clinical decision-making and optimize patient care.

Indeed, various studies have explored the prognostic significance of DLBCL patients and developed relevant prognostic models. For instance, Biccler et al. utilized a large dataset comprising clinicopathological and treatment data from the National Lymphoma Registry in Denmark and Sweden. They leveraged machine learning techniques and different survival models to construct a novel DLBCL prognosis model using the stacking method [20]. The quality of a machine learning model heavily relies on the quality of the training data. In the case of Biccler et al.’s study, the model was trained using data from the Nordic population. These data may not precisely reflect the characteristics of the Chinese population, making it challenging to directly apply the model to the Chinese context. In contrast, the data used in our study were obtained from a general cohort in southwest China, making it more representative of the overall DLBCL patient population in China. As a result, our nomogram model exhibits better generalizability and broader applicability in the Chinese setting. Furthermore, models based on machine learning algorithms often suffer from a “black box” effect, lacking proper interpretability. Clinicians may find it difficult to comprehend how the algorithm arrives at its conclusions. In contrast, the nomogram model developed in our study offers a clear and visual representation of the impact of different clinical variables on the outcome. This enhances interpretability, eliminating the issue of a “weak model interpretation.” By combining robust data from the Chinese population and providing a transparent and interpretable model, our nomogram model offers significant advantages over machine learning–based models, making it a valuable tool for clinicians in understanding and predicting the prognosis of DLBCL patients.

In our study, we employed LASSO regression and stepwise multivariate COX regression to screen a comprehensive range of clinical features and establish prognostic models using key clinical factors that are readily accessible in clinical practice. The results revealed that age played a pivotal role in patient prognosis, with the death group exhibiting a significantly higher median age than the survival group, likely due to the increased risk of concurrent comorbidities in older individuals. This emphasizes the importance of age as a critical factor affecting patient outcomes, consistent with previous findings by Kubota T et al. [30]. Notably, our nomogram model demonstrated that elevated levels of LDH were associated with higher mortality, reaffirming the significance of LDH as a prognostic indicator, which has been well established in studies such as that conducted by Sun et al. [31]. Similarly, β2-microglobulin, widely evaluated as a prognostic factor for hematological diseases, showed a consistent relationship with patient outcomes in our study. Studies from Japan, the USA, and South Korea have all indicated that elevated β2-microglobulin levels are predictive of poor progression-free survival (PFS) and overall survival (OS) in DLBCL patients receiving various treatments [6, 32–34]. Moreover, a large-scale study developed a new scoring system, “GELTAMO-IPI,” incorporating β2-microglobulin into NCCN-IPI, which better differentiates low-risk groups compared to IPI [35]. Incorporating laboratory parameters like CRP and LYM proved to be of higher and more stable prognostic value in our study. For instance, a study combining LDH and CRP to predict response and survival in newly diagnosed DLBCL patients showed promising results, indicating it as a useful clinical prognostic indicator [36]. Though the core mechanism for LYM’s impact on prognosis remains unclear, its inclusion in our study showed that increased LYM levels were associated with worse patient outcomes. Regarding B symptoms, their significance as valuable prognostic indicators was observed in our study, aligning with findings from previous research [37] [38]. Researchers identified B symptoms as an independent prognostic factor for reduced PFS and OS in DLBCL patients, and incorporating them in the prediction model significantly improved its predictive efficacy [38]. In conclusion, our study establishes a robust prognostic nomogram model for DLBCL by incorporating key clinical factors. The results highlight the importance of age, LDH, β2-microglobulin, CRP, LYM, and B symptoms as critical prognostic markers for guiding treatment decisions and improving patient outcomes in DLBCL.

This study adhered to standardized protocols and utilized reliable instruments, ensuring the reliability of data collection. Rigorous training processes were implemented to guarantee high-quality data collection by personnel. Being a cohort study, this design minimizes the impact of confounding factors, adding strength to the study. However, there are certain limitations that should be acknowledged. Firstly, the absence of external data for further validation and evaluation of the model is a limitation. External validation with data from different populations or settings would enhance the generalizability and robustness of the model. Secondly, the sample size of this study is relatively small, and larger prospective cohorts are required to validate and confirm the findings. A larger sample size would provide more statistical power and enhance the reliability of the results. Lastly, this study was conducted in a single center, which may limit the generalizability of the findings to other regions or populations. Variation in patient characteristics and treatment practices across different centers or regions could influence the performance and applicability of the prognostic model. Considering these limitations, further studies with external validation, larger sample sizes, and multicenter collaborations are warranted to strengthen the evidence and facilitate the wider applicability of the prognostic model in different populations and settings.

## Conclusions

We introduce a novel prognostic model for DLBCL patients that surpasses traditional DLBCL prognostic indicators by incorporating a broader range of easily accessible clinical parameters. This comprehensive approach significantly enhances the predictive accuracy and performance of the prognostic model. In contrast to relying solely on clinical trial data, this study employs a real cohort, which better represents the general population of lymphoma patients, making the model more applicable and widely relevant in routine clinical practice.

## Data Availability

The data that support the findings of this study are available from the corresponding author upon reasonable request.
